# Relationship between air temperature and dengue incidence: time series study in Minas Gerais, Brazil (2010-2019)

**DOI:** 10.1590/0102-311XPT076723

**Published:** 2024-03-25

**Authors:** João Pedro Medeiros Gomes, Igor Magaton Ribas, Pedro Augusto Rosa Valadares, Lucas Santos Jardim, Mário Círio Nogueira, Cássia de Castro Martins Ferreira, Aripuanã Sakurada Aranha Watanabe, Letícia de Castro Martins Ferreira

**Affiliations:** 1 Faculdade de Medicina, Universidade Federal de Juiz de Fora, Juiz de Fora, Brasil.; 2 Instituto de Ciências Humanas, Universidade Federal de Juiz de Fora, Juiz de Fora, Brasil.; 3 Instituto de Ciências Biológicas, Universidade Federal de Juiz de Fora, Juiz de Fora, Brasil.

**Keywords:** Dengue, Climate, Time Factors, Temperature, Dengue, Clima, Fatores de Tempo, Temperatura, Dengue, Clima, Factores de Tiempo, Temperatura

## Abstract

Air temperature is a climatic factor that affects the incidence of dengue, with effects varying according to time and space. We investigated the relationship between minimum air temperature and dengue incidence in Minas Gerais, Brazil, and evaluated the influence of socioeconomic and geographic variables on this relationship. This is a time series study with analysis conducted in three distinct stages: modeling using a distributed lag non-linear model, meta-analysis of models obtained, and meta-regression with geographic and socioeconomic data. Minimum temperature was a protective factor at extreme cold temperatures (RR = 0.65; 95%CI: 0.56-0.76) and moderate cold temperatures (RR = 0.71; 95%CI: 0.64-0.79), and a risk factor at moderate hot temperatures (RR = 1.15; 95%CI: 1.07-1.24), but not at extreme hot temperatures (RR = 1.1; 95%CI: 0.99-1.22). Heterogeneity of the models was high (I^2^ = 60%), which was also observed in meta-regression. Moderate and extreme cold temperatures have a protective effect, while moderate hot temperatures increase the risk. However, minimum air temperature does not explain the variability in the region, not even with the other variables in meta-regression.

## Resumo

A temperatura do ar é um fator climático que afeta a incidência da dengue, com efeitos variando conforme o tempo e o espaço. Investigamos a relação entre a temperatura mínima do ar e a incidência da doença em Minas Gerais, Brasil, e avaliamos a influência de variáveis socioeconômicas e geográficas nessa relação, calculando-se o risco relativo (RR). Este é um estudo de série temporal com análise conduzida em três etapas distintas: modelagem por uso de *distributed lag non-linear model* (modelos não-lineares distributivos com defasagem), metanálise dos modelos obtidos e metarregressão com dados geográficos e socioeconômicos. A temperatura mínima foi um fator de proteção quando em temperaturas frias extremas (RR = 0,65; IC95%: 0,56-0,76) e moderadas (RR = 0,71; IC95%: 0,64-0,79) e fator de risco em temperaturas de calor moderado (RR = 1,15; IC95%: 1,07-1,24), mas não em extremo (RR = 1,1; IC95%: 0,99-1,22). A heterogeneidade dos modelos foi elevada (I^2^ = 60%) e essa medida não foi alterada em metarregressão. Temperaturas frias moderadas e extremas causam efeito protetivo, enquanto moderadas quentes aumentam o risco. No entanto, a temperatura mínima do ar não explica nem a variabilidade da região, nem mesmo com as outras variáveis em metarregressão.

## Resumen

La temperatura del aire es un factor climático que afecta la incidencia del dengue, con efectos que varían según el tiempo y el territorio. Investigamos la relación entre la temperatura mínima del aire y la incidencia de la enfermedad en Minas Gerais, Brasil, y evaluamos la influencia de variables socioeconómicas y geográficas en esta relación. Se trata de un estudio de serie temporal cuyo análisis se realiza en tres etapas distintas: modelación mediante el uso de *distributed lag non-linear model* (modelos distributivos no lineales con retraso), metaanálisis de los modelos obtenidos y metarregresión con datos geográficos y socioeconómicos. La temperatura mínima fue un factor de protección ante temperaturas extremadamente frías (RR = 0,65; IC95%: 0,56-0,76) y moderadas (RR = 0,71; IC95%: 0,64-0,79) y factor de riesgo en temperaturas de calor moderado (RR = 1,15; IC95%: 1,07-1,24), pero no en extremo (RR = 1,1; IC95%: 0,99-1,22). La heterogeneidad de los modelos fue alta (I^2^ = 60%), y esta medida no se modificó en la metarregresión. Las temperaturas frías moderadas y extremas tienen un efecto protector, mientras que las temperaturas moderadamente altas aumentan el riesgo. Sin embargo, la temperatura mínima del aire no explica la variabilidad de la región, ni siquiera con las demás variables en metarregresión.

Este é um artigo publicado em acesso aberto (Open Access) sob a licença Creative Commons Attribution, que permite uso, distribuição e reprodução em qualquer meio, sem restrições, desde que o trabalho original seja corretamente citado.

## Introdução

A dengue é uma infecção arboviral com quatro possíveis sorotipos (DENV 1-4) e em constante crescimento, com 100 a 400 milhões de novas infecções por ano, transmitida pelo mosquito *Aedes aegypti* [1]. Ela é uma das principais doenças tropicais negligenciadas no mundo [2], sendo endêmica na maior parte do Brasil e com recorrentes epidemias no país [3]. Embora exista relação entre as faixas de latitude e a frequência dos casos de dengue no Brasil [4], a doença está se expandindo para além das faixas geográficas convencionais, tornando-se cada vez mais frequente nas regiões ao sul do país [5].

O Estado de Minas Gerais é o segundo mais populoso do país e apresenta um clima predominantemente tropical e temperado quente, mas com pequenas regiões ao norte com clima árido, segundo a classificação de Köppen [6]. Dessa maneira, abrange ambientes normalmente favoráveis à infestação de *A. aegypti* e, consequentemente, uma elevada incidência de dengue [7]. O resultado desse conjunto de fatores torna Minas Gerais o segundo com o maior número de casos de dengue no Brasil nos últimos dez anos, com recentes epidemias na sua capital, Belo Horizonte, bem como em outras cidades, resultando em um grande problema de saúde pública [8].

Fatores de riscos sociais, econômicos, demográficos e, principalmente, climáticos têm grande influência no comportamento epidemiológico da dengue [9]. Dessa maneira, densidade populacional, urbanização, mobilidade urbana, além de acesso a água e saneamento básico, são importantes variáveis que alteram o intercurso da arbovirose e de sua incidência [10]. Contudo, por serem fatores que caracteristicamente apresentarem mudanças lentas e graduais, impossibilitam a investigação de associações em intervalos curtos de tempo. Isso não se aplica aos fatores climáticos, em particular à temperatura do ar, que demonstra uma forte correlação com a dengue e seu vetor, sendo prontamente disponível em diversas escalas temporais. Isso permite uma análise detalhada e diária, contribuindo para uma compreensão mais precisa da magnitude e sazonalidade dessa doença [11].

Variáveis climáticas têm grande influência na taxa de desenvolvimento, na sobrevivência, no comportamento, na reprodução e na criação de ambientes propícios para o vetor da dengue, bem como na replicação viral, nos períodos de incubação e na transmissibilidade do vírus [10,12]. Essa influência está ligada a variáveis diversas, como índices de seca [10,13], de poluição [11], precipitação e seus padrões [11,14], além da temperatura do ar [12]. Essa última aparece como um dos fatores mais importantes, ao influenciar em todas as fases da vida do vetor e do ciclo do vírus, limitando, permitindo e controlando cada um de seus aspectos [12].

Dessa forma, embora a relação entre a temperatura do ar e a doença seja largamente explorada, carecem inferências quantitativas e abrangentes do efeito dessa variável em uma área como Minas Gerais, com grande número de municípios e heterogeneidade entre eles. Adicionalmente, o efeito de temperaturas do ar extremas, cada vez mais frequentes devido às mudanças climáticas, pode afetar a transmissão e as epidemias de dengue, modificando o padrão da doença e dificultando estratégias de intervenção no contexto de saúde coletiva [12,15]. Além disso, contribuem para a complexidade dessa relação os fatores humanos descritos anteriormente, como imunidade, influências socioeconômicas e comportamentais [12]. Portanto, entender como o tamanho do efeito da temperatura do ar em uma escala diária e se ater a influências socioeconômicas e geográficas pode gerar importantes informações para o planejamento de estratégias atuais e futuras para a prevenção e o entendimento da dinâmica da dengue.

Neste trabalho, visamos contribuir na complexa investigação da relação diária, não linear e em defasagem, da associação entre casos de dengue e a temperatura do ar sobre as microrregiões do Estado de Minas Gerais. Dessa maneira, este é um estudo de série temporal no qual descrevemos a distribuição dos casos de dengue em todas as microrregiões de Minas Gerais e analisamos a relação entre a temperatura mínima do ar e a incidência dessa doença na maior parte dessas microrregiões. Em conjunto, também avaliamos essa relação com outras variáveis socioeconômicas e geográficas.

## Metodologia

### Área de estudo e dados da dengue

Minas Gerais é o segundo estado mais populoso do Brasil, com uma população estimada em 21.411.923 pessoas em 2021 [16]. De acordo com a divisão geográfica regional de Minas Gerais, vigente na maior parte do período do estudo, o estado contém 12 mesorregiões subdivididas em 66 microrregiões – grupos de municípios limítrofes com características socioeconômicas comuns – com 853 municípios no total [8,16].

Entre as 66 microrregiões do estado, apenas 38 foram selecionadas para a modelagem deste estudo, segundo o critério de inclusão estabelecido. Sul e Sudoeste de Minas, Vale do Rio Doce e Jequitinhonha foram as mesorregiões com mais microrregiões excluídas, seis, cinco e cinco, respectivamente. Apenas uma microrregião foi incluída nas mesorregiões de Campo das Vertentes e Vale do Mucuri, enquanto Jequitinhonha foi totalmente excluída. As mesorregiões da Zona da Mata e do Triângulo Mineiro e Alto Paranaíba apresentaram o maior número de microrregiões, ambas com sete. As demais mesorregiões tiveram entre duas e cinco microrregiões incluídas (Figura 1).

Os bancos de microdados de notificação de dengue do Sistema de Informação de Agravos de Notificação (SINAN) para o período de 2013-2019 foram obtidos no Departamento de Informática do SUS (DATASUS); para o período de 2010-2012, foram solicitados ao Ministério da Saúde via Lei de Acesso à Informação [17]. Notificações identificadas oficialmente como casos duplicados ou descartados foram excluídas. A seguir, os dados foram tabulados no programa R (http://www.r-project.org) por frequência diária de casos por microrregião, entre o início de 2010 e o último dia de 2019. Esse período foi utilizado tendo em vista que análises exploratórias demonstraram maior consistência e qualidade dos dados. Informações referentes a todas as microrregiões foram utilizadas para a análise descritiva. Contudo, para inclusão na modelagem, a partir de critérios metodológicos e análise exploratória, foi estabelecido uma quantidade mínima de 10 mil casos no período analisado, já que a elevada frequência de dias sem casos, com variabilidade aleatória maior, levaria a estimativas altamente imprecisas ou erros de iteração, prejudicando a confiabilidade da metanálise subsequente.

### Dados meteorológicos, geográficos e socioeconômicos

Dados de temperatura do ar foram obtidos por meio do modelo de reanálise ERA5 [18], dados diários de variáveis meteorológicas agregados a partir de um modelo horário construído pelo Centro Europeu de Previsões Meteorológicas a Médio Prazo (ECMWF; European Centre for Medium-Range Weather Forecasts) que apresenta um *dataset* completo e consistente, gerado a partir de observações e com modelagem baseada nas leis da Física, tendo resolução espacial de 0,1º [18]. Nesse caso, esses dados foram agregados em média para cada microrregião. Foram extraídos dados de coordenadas dos centroides e da altitude média de cada microrregião, a partir do uso das bibliotecas do programa R, *terra* e *elevatr* (https://zenodo.org/record/5809645). Para essa análise, as temperaturas dos percentis p2,5, p10, p90 e p97,5 foram consideradas, respectivamente, como extremamente frias, moderadamente frias, moderadamente quentes e extremamente quentes.

Dados referentes ao acesso a saneamento básico e coleta de lixo e à urbanização foram obtidos a partir do Instituto Brasileiro de Geografia e Estatística (IBGE) por meio do DATASUS, sendo referentes ao *Censo Demográfico* de 2010. Produto interno bruto (PIB), população e PIB *per capita* foram adquiridos a partir de estimativas anuais do IBGE [19].

As variáveis na metarregressão foram adicionadas por sua fácil disponibilidade e alta qualidade na área de estudo, além de relações com o desfecho encontradas em estudos prévios [9,10,20,21].

### Análise estatística

Este foi um estudo agregado e ecológico de análise de séries temporais realizado em três etapas: (1) uso conjunto de um modelo aditivo generalizado (GAM; *generalized additive model*) e um *modelos não-lineares distributivos com defasagem* (*DLNM; distributed lag non-linear model*) para estimar a associação em cada microrregião; (2) metanálise dos modelos para a síntese da relação da temperatura mínima do ar

**Figura 1**

Mesorregiões e microrregiões de Minas Gerais, Brasil, e microrregiões incluídas em metanálise.

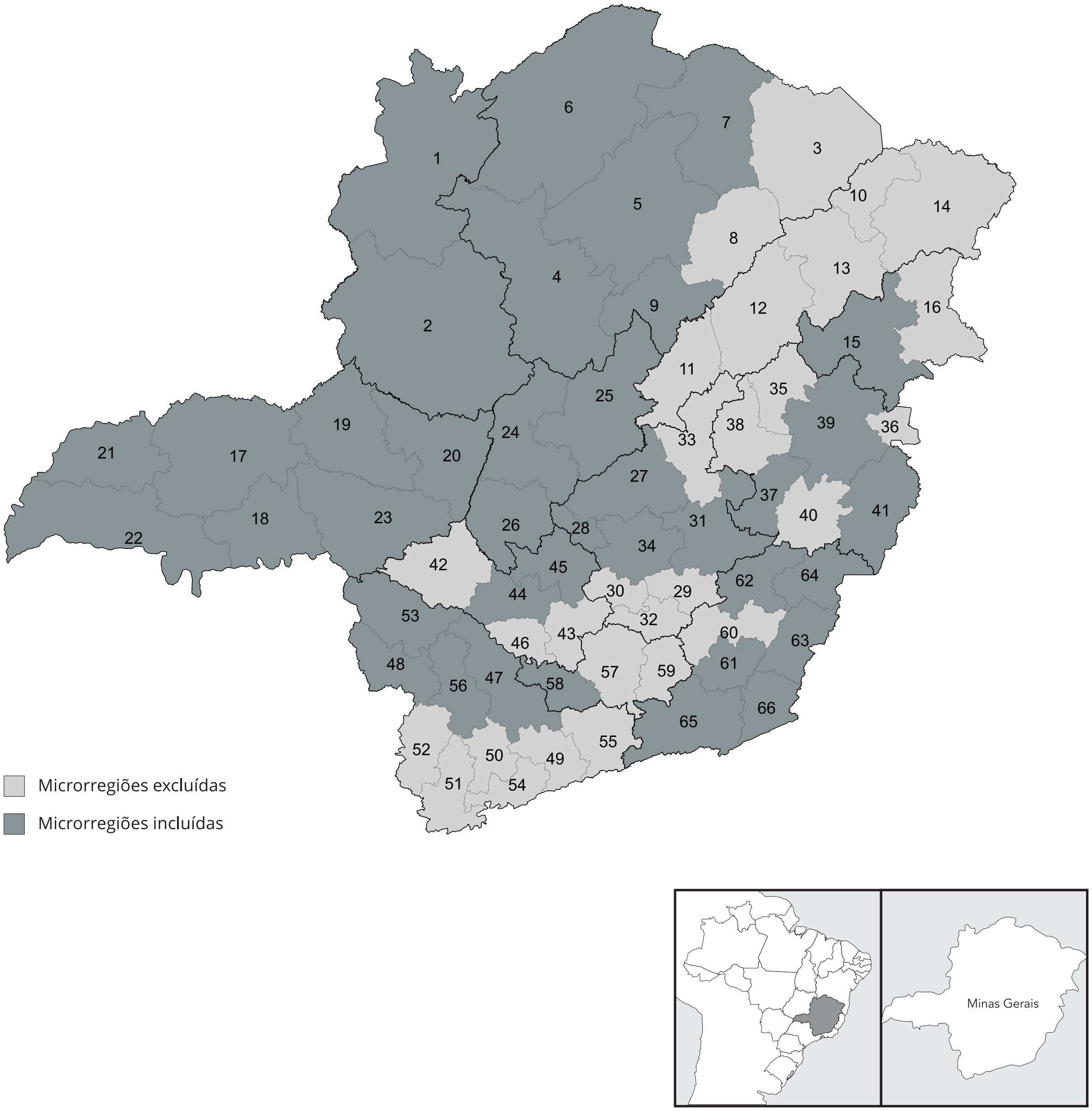

Fonte: Instituto Brasileiro de Geografia e Estatística [16].

Microrregiões: Mesorregião Noroeste de Minas (1. Unaí, 2. Paracatu); Mesorregião Norte de Minas (3. Salinas, 4. Pirapora, 5. Montes Claros, 6. Januária, 7. Janaúba, 8. Grão Mogol, 9. Bocaiúva); Jequitinhonha (10. Pedra Azul, 11. Diamantina, 12. Capelinha, 13. Araçuaí, 14. Almenara); Mesorregião Vale do Mucuri (15. Teófilo Otoni, 16. Nanuque); Mesorregião Triângulo Mineiro e Alto Paranaíba (17. Uberlândia, 18. Uberaba, 19. Patrocínio, 20. Patos de Minas, 21. Ituiutaba, 22. Frutal, 23. Araxá); Mesorregião Central Mineira (24. Três Marias, 25. Curvelo, 26. Bom Despacho); Mesorregião Metropolitana de Belo Horizonte (27. Sete Lagoas, 28. Pará de Minas, 29. Ouro Preto, 30. Itaguara, 31. Itabira, 32. Conselheiro Lafaiete, 33. Conceição do Mato Dentro, 34. Belo Horizonte); Mesorregião Vale do Rio Doce (35. Peçanha, 36. Mantena, 37. Ipatinga, 38. Guanhães, 39. Governador Valadares, 40. Caratinga, 41. Aimorés); Mesorregião Oeste de Minas (42. Piuí, 43. Oliveira, 44. Formiga, 45. Divinópolis, 46. Campo Belo, 47. Varginha); Mesorregião Sul e Sudoeste de Minas (48. São Sebastião do Paraíso, 49. São Lourenço, 50. Santa Rita do Sapucaí, 51. Pouso Alegre, 52. Poços de Caldas, 53. Passos, 54. Itajubá, 55. Andrelândia, 56. Alfenas); Mesorregião Campo das Vertentes (57. São João Del Rei, 58. Lavras, 59. Barbacena); Mesorregião Zona da Mata (60. Viçosa, 61. Ubá, 62. Ponte Nova, 63. Muriaé, 64. Manhuaçu, 65. Juiz de Fora, 66. Cataguases).

e os casos de dengue; (3) metarregressão com variáveis geográficas e socioeconômicas que poderiam ser explicativas quanto à heterogeneidade nas medidas de associação. Todas as análises foram realizadas no software estatístico R e foi estabelecido um intervalo de 95% de confiança (IC95%).

- **Etapa 1: GAM e DLNM**

Foi utilizada uma aplicação em conjunto de um GAM e um DLNM, na qual a temperatura mínima do ar era a variável preditiva. Essa temperatura foi escolhida com base em estudos anteriores que demonstraram essa variável como melhor preditor quando comparado com as temperaturas médias e máximas [10,13,22,23]. Para a construção do modelo, foi feita uma seleção sistemática dos parâmetros do modelo com base na menor soma dos critérios de informação de Akaike (AIC; *Akaike information criterion*) das microrregiões em cada variação de modelagem [10,24]. Essa seleção foi realizada por uma eliminação seriada em que primeiramente foram selecionados os graus de liberdade do *spline* do tempo, seguidos do intervalo da defasagem a ser analisado e, por fim, da introdução da população como *offset*.

O modelo final teve como parâmetros: um *crossbasis* de 25 dias de *lag* da temperatura mínima do ar como variável preditiva; um *spline* do tempo de 8 graus de liberdade por ano para controle de fatores temporais de longo prazo; os dias da semana como controle para variações de curto prazo; e, devido a uma diferença de AIC desprezível, a população como *offset* não foi incluída. Como medida de comparação usada para a construção do risco relativo (RR), foi utilizada a média da temperatura mínima de todas as regiões incluídas.

- **Etapa 2: metanálise**

Os modelos gerados pela primeira etapa passaram por uma redução de dimensionalidade conforme Gasparrini et al. [24]. Foi utilizada a biblioteca *mvmeta* para construir a metanálise de efeitos aleatórios. Esse tipo de modelo foi escolhido a partir da heterogeneidade do efeito da temperatura sobre as diversas microrregiões e o objetivo de analisar o seu efeito sobre a maior parte do estado [25,26]. Em seguida, foi feita a análise da curva do RR acumulado, dos riscos em cada uma das temperaturas moderadas e extremas e da heterogeneidade dos resultados, por meio do resultado da medida estatística $I^2$ [27].

- **Etapa 3: metarregressão**

Tendo em vista a alta heterogeneidade dos resultados, variáveis geográficas (latitude e longitude dos centroides e altitude média) e socioeconômicas (acesso à saneamento básico, acesso à coleta de lixo, urbanização, PIB, população e PIB *per capita*) foram introduzidas uma a uma no modelo final. Foi realizado o teste de Wald para avaliar se a introdução de uma dessas novas variáveis como efeito fixo levaria à diminuição da heterogeneidade do modelo. Resultados significantes nesse teste foram introduzidos em uma metarregressão e os percentis 10 e 90 da variável foram utilizados para demonstrar seus efeitos sobre a metanálise final [24,28].

## Resultados

### Análise descritiva dos dados de casos

As 66 microrregiões de Minas Gerais totalizaram 2.016.686 de casos no intervalo de 2010 a 2019. Nessas microrregiões, a média de casos diários variou de próxima a 0 até 244. A cidade com maior número de casos (891.462) e também maior máxima diária (5.691) foi Belo Horizonte. Dados relativos à distribuição de casos em cada microrregião podem ser encontrados na Tabela 1.

**Tabela 1**

Distribuição dos casos diários de dengue nas microrregiões de Minas Gerais, Brasil, 2010-2019.

| Microrregiões | População média * | Casos de dengue | | | Distribuição diária | | | | | | |
|---|---|---|---|---|---|---|---|---|---|---|---|
| | | Total | Média anual | Taxa média anual ** | Valor mínimo | Percentil 25 | Mediana | Média | Percentil 75 | Valor máximo | DP |
| Aimorés | 152.580 | 11.803 | 1.180 | 773,36 | 0 | 0 | 0 | 3,23 | 3 | 63 | 6,96 |
| Alfenas | 235.479 | 10.143 | 1.014 | 430,61 | 0 | 0 | 0 | 2,78 | 1 | 98 | 9,15 |
| Almenara | 185.494 | 3.551 | 355 | 191,38 | 0 | 0 | 0 | 0,97 | 1 | 64 | 3,52 |
| Andrelândia | 73.336 | 234 | 23 | 31,36 | 0 | 0 | 0 | 0,06 | 0 | 7 | 0,39 |
| Araçuaí | 160.943 | 7.879 | 788 | 489,61 | 0 | 0 | 0 | 2,16 | 1 | 98 | 6,83 |
| Araxá | 219.192 | 14.979 | 1.498 | 683,42 | 0 | 0 | 1 | 4,10 | 3 | 86 | 9,52 |
| Barbacena | 223.836 | 4.301 | 430 | 192,10 | 0 | 0 | 0 | 1,18 | 0 | 124 | 6,47 |
| Belo Horizonte | 5.044.354 | 891.462 | 89.146 | 1.767,24 | 0 | 4 | 11 | 244,10 | 54 | 5691 | 710,24 |
| Bocaiúva | 71.833 | 10.933 | 1.093 | 1.521,58 | 0 | 0 | 0 | 2,99 | 1 | 135 | 10,10 |
| Bom Despacho | 174.138 | 25.120 | 2.512 | 1.442,53 | 0 | 0 | 1 | 6,88 | 4 | 212 | 18,40 |
| Campo Belo | 116.055 | 8.273 | 827 | 712,59 | 0 | 0 | 0 | 2,27 | 0 | 93 | 8,26 |
| Capelinha | 204.770 | 8.582 | 858 | 419,01 | 0 | 0 | 0 | 2,35 | 1 | 117 | 8,19 |
| Caratinga | 262.736 | 8.397 | 840 | 319,71 | 0 | 0 | 0 | 2,30 | 1 | 102 | 8,90 |
| Cataguases | 223.886 | 19.357 | 1.936 | 864,73 | 0 | 0 | 0 | 5,30 | 1 | 160 | 16,75 |
| Conceição do Mato Dentro | 83.040 | 1.889 | 189 | 227,60 | 0 | 0 | 0 | 0,52 | 0 | 31 | 2,00 |
| Conselheiro Lafaiete | 261.489 | 4.571 | 457 | 174,77 | 0 | 0 | 0 | 1,25 | 0 | 63 | 4,96 |
| Curvelo | 155.991 | 27.729 | 2.773 | 1.777,67 | 0 | 0 | 1 | 7,59 | 4 | 239 | 21,36 |
| Diamantina | 84.548 | 4.208 | 421 | 497,94 | 0 | 0 | 0 | 1,15 | 0 | 87 | 4,80 |
| Divinópolis | 525.211 | 62.892 | 6.289 | 1.197,42 | 0 | 0 | 1 | 17,22 | 7 | 435 | 43,38 |
| Formiga | 157.558 | 20.961 | 2.096 | 1.330,30 | 0 | 0 | 0 | 5,74 | 2 | 141 | 15,77 |
| Frutal | 191.894 | 20.521 | 2.052 | 1.069,34 | 0 | 0 | 1 | 5,62 | 4 | 183 | 13,28 |
| Governador Valadares | 430.050 | 28.902 | 2.890 | 672,01 | 0 | 0 | 2 | 7,91 | 8 | 143 | 15,29 |
| Grão Mogol | 44.170 | 1.658 | 166 | 375,82 | 0 | 0 | 0 | 0,45 | 0 | 22 | 1,77 |
| Guanhães | 134.379 | 2.231 | 223 | 165,95 | 0 | 0 | 0 | 0,61 | 0 | 29 | 2,13 |
| Ipatinga | 555.433 | 70.244 | 7.024 | 1.264,60 | 0 | 1 | 4 | 19,23 | 12 | 846 | 60,68 |
| Itabira | 395.688 | 21.157 | 2.116 | 534,76 | 0 | 0 | 0 | 5,79 | 2 | 254 | 17,77 |
| Itaguara | 63.736 | 4.500 | 450 | 706,04 | 0 | 0 | 0 | 1,23 | 0 | 49 | 4,33 |
| Itajubá | 193.683 | 2.523 | 252 | 130,11 | 0 | 0 | 0 | 0,69 | 0 | 73 | 4,67 |
| Ituiutaba | 149.676 | 19.015 | 1.902 | 1.270,74 | 0 | 0 | 1 | 5,21 | 5 | 92 | 10,15 |
| Janaúba | 255.399 | 17.588 | 1.759 | 688,73 | 0 | 0 | 0 | 4,82 | 3 | 167 | 12,72 |
| Januária | 285.299 | 15.454 | 1.545 | 541,54 | 0 | 0 | 1 | 4,23 | 3 | 148 | 10,83 |
| Juiz de Fora | 765.100 | 77.098 | 7.710 | 1.007,71 | 0 | 0 | 1 | 21,11 | 5 | 913 | 69,02 |
| Lavras | 157.806 | 11.747 | 1.175 | 744,59 | 0 | 0 | 0 | 3,22 | 1 | 196 | 13,47 |
| Manhuaçu | 287.269 | 11.097 | 1.110 | 386,40 | 0 | 0 | 0 | 3,04 | 1 | 97 | 10,68 |
| Mantena | 64.854 | 3.743 | 374 | 576,68 | 0 | 0 | 0 | 1,02 | 1 | 55 | 3,20 |
| Montes Claros | 635.459 | 47.150 | 4.715 | 741,98 | 0 | 0 | 1 | 12,91 | 6 | 498 | 34,95 |
| Muriaé | 286.260 | 17.257 | 1.726 | 602,95 | 0 | 0 | 0 | 4,73 | 2 | 159 | 13,59 |
| Nanuque | 120.577 | 7.130 | 713 | 591,32 | 0 | 0 | 0 | 1,95 | 1 | 53 | 5,63 |
| Oliveira | 130.650 | 4.026 | 403 | 308,46 | 0 | 0 | 0 | 1,10 | 0 | 42 | 3,62 |
| Ouro Preto | 183.791 | 2.782 | 278 | 151,26 | 0 | 0 | 0 | 0,76 | 0 | 46 | 2,78 |
| Pará de Minas | 131.500 | 14.056 | 1.406 | 1.069,20 | 0 | 0 | 0 | 3,85 | 1 | 151 | 13,46 |
| Paracatu | 229.068 | 28.243 | 2.824 | 1232,82 | 0 | 0 | 0 | 7,73 | 4 | 146 | 18,50 |

(continua)

**Tabela 1 (continuação)**

| Microrregiões | População média * | Casos de dengue | | | Distribuição diária | | | | | | |
|---|---|---|---|---|---|---|---|---|---|---|---|
| | | Total | Média anual | Taxa média anual ** | Valor mínimo | Percentil 25 | Mediana | Média | Percentil 75 | Valor máximo | DP |
| Passos | 236.387 | 18.353 | 1.835 | 776,27 | 0 | 0 | 0 | 5,03 | 3 | 129 | 11,78 |
| Patos de Minas | 265.420 | 24.675 | 2.468 | 929,85 | 0 | 0 | 1 | 6,76 | 4 | 204 | 18,66 |
| Patrocínio | 206.306 | 13.967 | 1.397 | 677,15 | 0 | 0 | 0 | 3,82 | 2 | 138 | 11,48 |
| Peçanha | 82.001 | 2.228 | 223 | 271,95 | 0 | 0 | 0 | 0,61 | 0 | 30 | 2,12 |
| Pedra Azul | 83.898 | 4.309 | 431 | 513,72 | 0 | 0 | 0 | 1,18 | 0 | 91 | 4,75 |
| Pirapora | 171.568 | 16.524 | 1.652 | 962,88 | 0 | 0 | 0 | 4,52 | 1 | 167 | 13,56 |
| Piumhi | 84.724 | 5.246 | 525 | 619,66 | 0 | 0 | 0 | 1,44 | 1 | 52 | 4,43 |
| Poços de Caldas | 359.702 | 2.104 | 210 | 58,38 | 0 | 0 | 0 | 0,58 | 0 | 60 | 2,93 |
| Ponte Nova | 189.490 | 10.176 | 1.018 | 537,23 | 0 | 0 | 0 | 2,79 | 1 | 90 | 8,09 |
| Pouso Alegre | 346.805 | 1.354 | 135 | 38,93 | 0 | 0 | 0 | 0,37 | 0 | 35 | 1,99 |
| Salinas | 218.594 | 4.946 | 495 | 226,45 | 0 | 0 | 0 | 1,35 | 0 | 60 | 4,25 |
| Santa Rita do Sapucaí | 144.263 | 2.253 | 225 | 155,97 | 0 | 0 | 0 | 0,62 | 0 | 36 | 2,71 |
| São João Del Rei | 190.195 | 5.136 | 514 | 270,25 | 0 | 0 | 0 | 1,41 | 0 | 62 | 4,97 |
| São Lourenço | 213.925 | 3.378 | 338 | 158,00 | 0 | 0 | 0 | 0,92 | 0 | 65 | 4,27 |
| São Sebastião do Paraíso | 275.155 | 10.956 | 1.096 | 398,32 | 0 | 0 | 0 | 3,00 | 1 | 79 | 7,94 |
| Sete Lagoas | 418.140 | 70.962 | 7.096 | 1.697,04 | 0 | 0 | 1 | 19,43 | 4 | 742 | 65,67 |
| Teófilo Otoni | 273.630 | 24.635 | 2.464 | 900,49 | 0 | 0 | 1 | 6,75 | 4 | 360 | 21,61 |
| Três Marias | 101.569 | 13.804 | 1.380 | 1.358,68 | 0 | 0 | 0 | 3,78 | 2 | 107 | 10,14 |
| Ubá | 284.647 | 35.396 | 3.540 | 1.243,65 | 0 | 0 | 1 | 9,69 | 5 | 471 | 30,78 |
| Uberaba | 372.247 | 32.196 | 3.220 | 865,02 | 0 | 0 | 1 | 8,82 | 7 | 313 | 25,37 |
| Uberlândia | 877.730 | 91.373 | 9.137 | 1.040,98 | 0 | 1 | 4 | 25,02 | 15 | 604 | 62,64 |
| Unaí | 155.549 | 21.858 | 2.186 | 1.405,34 | 0 | 0 | 1 | 5,99 | 3 | 162 | 16,04 |
| Varginha | 461.461 | 19.035 | 1.904 | 412,60 | 0 | 0 | 0 | 5,21 | 3 | 137 | 14,04 |
| Viçosa | 227.918 | 64.36 | 644 | 282,56 | 0 | 0 | 0 | 1,76 | 0 | 149 | 8,28 |

DP: desvio padrão.
* Média da população do período;
** Taxas por 100.000 habitantes.

É possível perceber um aspecto cíclico, já que em anos diferentes temos picos de incidência em diferentes magnitudes (Figura 2a) e sazonal, devido à concentração de casos entre os primeiros cinco meses do ano (Figura 2b). Outro ponto a ser ressaltado é a distribuição de casos por dia da semana (Figura 2c). A grande concentração de casos nos dias úteis é um fato esperado, já que, nos finais de semana, é comum o menor funcionamento do sistema de saúde. Esse fenômeno justifica a introdução dos dias da semana como uma maneira de controlar efeitos a curto prazo no processo de modelagem diária. Quando avaliada a distribuição dos casos por meses em cada ano e microrregiões, é possível notar o caráter cíclico das epidemias de dengue, que, nos dados apresentados, tornam-se mais intensas a cada três anos, aproximadamente, bem como há maior notificação de casos nos meses de dezembro a junho (Figura 2d).

## Análise descritiva da temperatura mínima

A distribuição da temperatura mínima também se mostrou bastante heterogênea, sendo que a menor temperatura mínima do ar foi de 2,5ºC em Pouso Alegre e a maior foi de 26,5ºC em Frutal e Ituiutaba. A região sul do estado apresentou as menores médias de temperaturas mínimas, as mesorregiões Sul e Sudoeste de Minas, Campo das Vertentes e Metropolitana de Belo Horizonte apresentaram as

**Figura 2**

Distribuição da soma de casos diária, mensal, por dia da semana e da incidência por mês e ano.

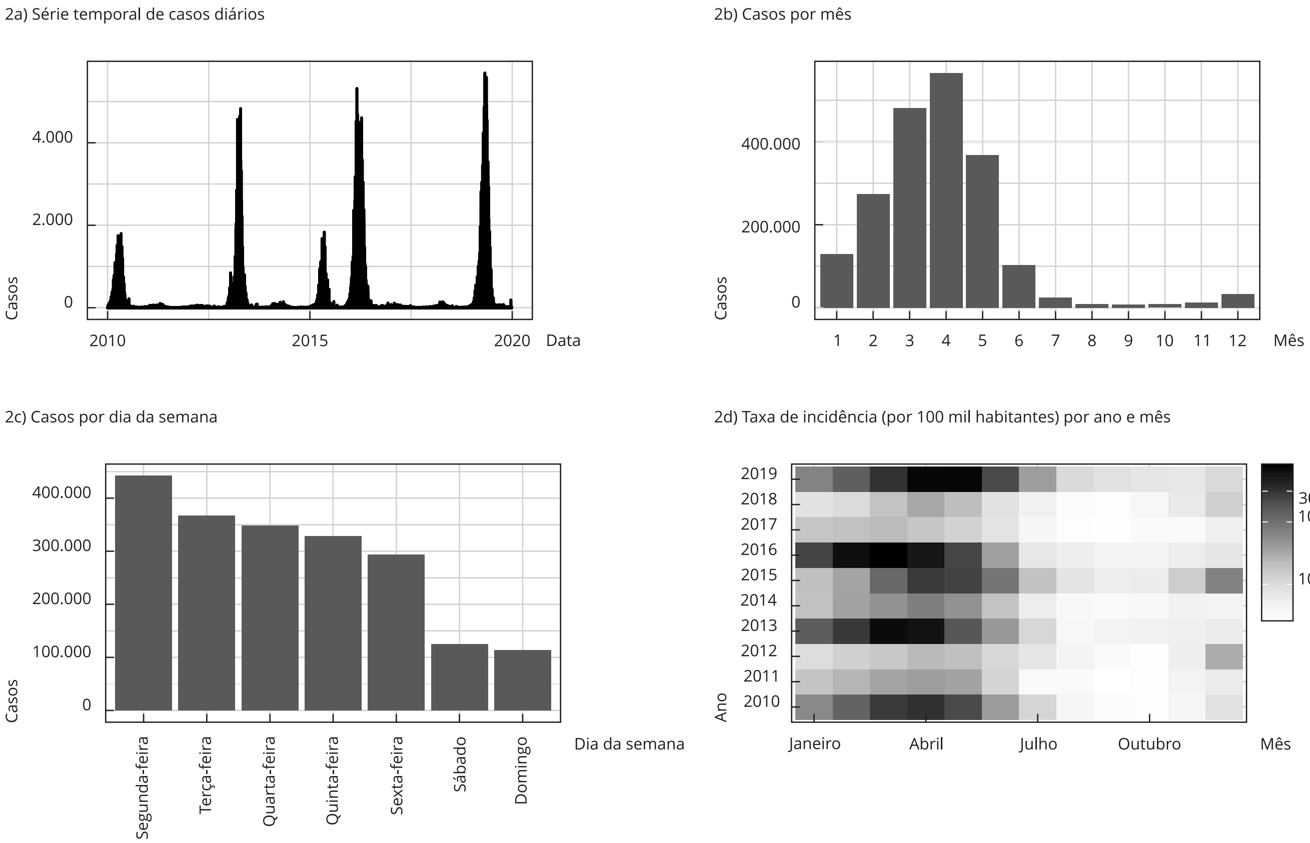

microrregiões com as menores médias de temperaturas mínimas, principalmente: Itajubá (13,6ºC), Barbacena (14,8ºC) e Conselheiro Lafaiete (15ºC), respectivamente. Em contrapartida, as mesorregiões do Triângulo Mineiro e Alto Paranaíba, Norte de Minas e Vale do Rio Doce destacam-se pelas microrregiões com as médias de temperaturas mínimas mais elevadas. Elas têm como principais representantes, nessa ordem, as microrregiões de Ituiutaba (20,4ºC), Januária (20,2ºC) e Governador Valadares (19,7ºC) (Tabela 2; Figura 3).

## Metanálise

A metanálise de efeitos aleatórios das 38 microrregiões revelou a temperatura mínima como um fator de proteção quando em temperaturas frias extremas (RR = 0,65; IC95%: 0,56-0,76) e moderadas (RR = 0,71; IC95%: 0,64-0,79) e fator de risco em temperaturas de calor moderado (RR = 1,15; IC95%: 1,07-1,24), mas não em extremo (RR = 1,1; IC95%: 0,99-1,22) (Tabela 3; Figura 4a). A partir da estatística $I^2$, foi percebida grande heterogeneidade nos modelos ($I^2$ = 60%).

**Tabela 2**

Distribuição dos dados diários de temperatura mínima média (em ºC) nas microrregiões estudadas, Minas Gerais, Brasil, 2010-2019.

| Microrregiões | Valor mínimo | Percentil 2,5 | Percentil 10 | Mediana | Média | Percentil 75 | Percentil 90 | Percentil 97,5 | Valor máximo |
|---|---|---|---|---|---|---|---|---|---|
| Aimorés | 10,4 | 14,0 | 15,6 | 19,5 | 19,0 | 21,0 | 21,8 | 22,6 | 24,1 |
| Alfenas | 6,1 | 10,0 | 11,9 | 16,9 | 16,2 | 18,7 | 19,5 | 20,0 | 21,7 |
| Almenara | 12,2 | 14,5 | 15,9 | 19,0 | 18,6 | 20,1 | 20,9 | 21,6 | 23,0 |
| Andrelândia | 4,6 | 7,9 | 9,8 | 14,4 | 13,9 | 16,3 | 17,2 | 17,8 | 18,9 |
| Araçuaí | 11,5 | 13,5 | 15,2 | 18,7 | 18,2 | 19,9 | 20,6 | 21,4 | 23,3 |
| Araxá | 6,4 | 11,1 | 12,9 | 17,4 | 16,6 | 18,5 | 19,2 | 19,8 | 22,4 |
| Barbacena | 6,0 | 9,2 | 10,9 | 15,2 | 14,8 | 17,1 | 18,0 | 18,6 | 19,7 |
| Belo Horizonte | 7,6 | 10,9 | 12,4 | 16,9 | 16,2 | 18,5 | 19,3 | 20,0 | 21,0 |
| Bocaiúva | 10,3 | 12,9 | 14,3 | 18,4 | 17,8 | 19,6 | 20,4 | 21,1 | 23,1 |
| Bom Despacho | 8,3 | 11,8 | 13,6 | 18,5 | 17,7 | 19,9 | 20,7 | 21,5 | 23,9 |
| Campo Belo | 7,0 | 10,2 | 12,1 | 16,9 | 16,3 | 18,7 | 19,5 | 20,1 | 21,7 |
| Capelinha | 10,1 | 12,6 | 14,1 | 18,0 | 17,4 | 19,2 | 19,9 | 20,7 | 22,5 |
| Caratinga | 9,2 | 13,3 | 14,7 | 18,7 | 18,2 | 20,2 | 21,1 | 21,8 | 23,2 |
| Cataguases | 10,5 | 12,9 | 14,8 | 19,0 | 18,6 | 20,9 | 21,8 | 22,5 | 23,9 |
| Conceição do Mato Dentro | 7,9 | 11,0 | 12,5 | 16,8 | 16,1 | 18,2 | 18,9 | 19,7 | 21,1 |
| Conselheiro Lafaiete | 6,3 | 9,4 | 11,0 | 15,6 | 15,0 | 17,5 | 18,3 | 19,0 | 19,8 |
| Curvelo | 10,2 | 13,3 | 14,8 | 19,1 | 18,4 | 20,4 | 21,1 | 22,0 | 24,2 |
| Diamantina | 8,0 | 11,1 | 12,5 | 16,8 | 16,1 | 18,1 | 18,8 | 19,5 | 21,3 |
| Divinópolis | 7,5 | 11,0 | 12,8 | 17,5 | 16,8 | 19,1 | 19,9 | 20,6 | 22,8 |
| Formiga | 7,7 | 10,9 | 12,7 | 17,4 | 16,7 | 19,0 | 19,8 | 20,4 | 21,9 |
| Frutal | 7,7 | 13,7 | 16,6 | 21,0 | 20,2 | 22,1 | 22,8 | 23,8 | 26,5 |
| Governador Valadares | 11,2 | 14,6 | 16,4 | 20,1 | 19,7 | 21,5 | 22,3 | 23,2 | 25,0 |
| Grão Mogol | 10,4 | 12,7 | 14,1 | 17,9 | 17,3 | 19,0 | 19,8 | 20,5 | 23,3 |
| Guanhães | 8,8 | 12,0 | 13,5 | 17,6 | 17,0 | 19,0 | 19,7 | 20,5 | 22,1 |
| Ipatinga | 10,0 | 13,5 | 14,9 | 18,9 | 18,4 | 20,4 | 21,2 | 21,9 | 23,3 |
| Itabira | 7,4 | 11,6 | 13,0 | 17,2 | 16,7 | 18,8 | 19,6 | 20,3 | 21,3 |
| Itaguara | 7,1 | 10,1 | 11,7 | 16,2 | 15,6 | 17,9 | 18,7 | 19,3 | 20,3 |
| Itajubá | 3,3 | 7,4 | 9,6 | 14,2 | 13,6 | 16,0 | 16,9 | 17,6 | 18,7 |
| Ituiutaba | 7,7 | 14,1 | 17,1 | 21,1 | 20,4 | 22,1 | 22,8 | 23,8 | 26,5 |
| Janaúba | 13,6 | 15,6 | 17,0 | 20,3 | 19,9 | 21,3 | 22,1 | 23,1 | 25,6 |
| Januária | 13,5 | 16,0 | 17,4 | 20,6 | 20,2 | 21,6 | 22,4 | 23,3 | 26,3 |
| Juiz de Fora | 6,5 | 10,4 | 12,3 | 16,6 | 16,2 | 18,5 | 19,5 | 20,2 | 21,4 |
| Lavras | 5,9 | 8,9 | 10,9 | 15,8 | 15,2 | 17,6 | 18,5 | 19,1 | 20,1 |
| Manhuaçu | 7,5 | 11,1 | 12,7 | 16,6 | 16,2 | 18,3 | 19,1 | 19,9 | 21,0 |
| Mantena | 11,6 | 13,9 | 15,8 | 19,3 | 18,9 | 20,6 | 21,4 | 22,1 | 23,5 |
| Montes Claros | 12,4 | 15,1 | 16,3 | 19,7 | 19,3 | 20,8 | 21,7 | 22,8 | 25,9 |
| Muriaé | 8,2 | 11,3 | 13,0 | 17,3 | 16,8 | 19,2 | 20,1 | 20,8 | 22,2 |
| Nanuque | 12,6 | 15,1 | 16,6 | 20,1 | 19,7 | 21,3 | 22,3 | 23,2 | 24,8 |
| Oliveira | 6,7 | 9,6 | 11,5 | 16,2 | 15,5 | 17,9 | 18,7 | 19,3 | 20,6 |
| Ouro Preto | 6,0 | 10,0 | 11,5 | 15,9 | 15,3 | 17,7 | 18,5 | 19,1 | 19,9 |
| Pará de Minas | 7,9 | 11,7 | 13,4 | 17,9 | 17,2 | 19,4 | 20,2 | 21,2 | 23,6 |
| Paracatu | 10,7 | 14,3 | 15,7 | 19,7 | 19,0 | 20,7 | 21,4 | 22,5 | 25,1 |
| Passos | 6,9 | 11,3 | 13,1 | 17,9 | 17,1 | 19,3 | 20,0 | 20,6 | 22,7 |
| Patos de Minas | 8,0 | 11,7 | 13,4 | 17,8 | 17,0 | 18,9 | 19,6 | 20,4 | 22,4 |

(continua)

**Tabela 2 (continuação)**

| Microrregiões | Valor mínimo | Percentil 2,5 | Percentil 10 | Mediana | Média | Percentil 75 | Percentil 90 | Percentil 97,5 | Valor máximo |
|---|---|---|---|---|---|---|---|---|---|
| Patrocínio | 8,1 | 12,5 | 14,1 | 18,3 | 17,5 | 19,3 | 20,0 | 20,8 | 23,3 |
| Peçanha | 10,4 | 13,2 | 14,9 | 18,8 | 18,3 | 20,1 | 21,0 | 21,8 | 23,5 |
| Pedra Azul | 11,4 | 13,6 | 15,2 | 18,4 | 18,0 | 19,5 | 20,2 | 20,9 | 23,2 |
| Pirapora | 12,5 | 15,1 | 16,5 | 20,2 | 19,7 | 21,3 | 22,2 | 23,3 | 25,7 |
| Piumhi | 6,7 | 10,7 | 12,5 | 17,3 | 16,4 | 18,7 | 19,5 | 20,1 | 21,9 |
| Poços de Caldas | 3,5 | 8,4 | 10,8 | 15,6 | 14,8 | 17,3 | 18,0 | 18,7 | 20,0 |
| Ponte Nova | 8,2 | 12,3 | 13,9 | 18,4 | 17,8 | 20,1 | 21,0 | 21,7 | 22,9 |
| Pouso Alegre | 2,5 | 7,7 | 10,2 | 14,8 | 14,1 | 16,6 | 17,4 | 18,1 | 19,2 |
| Salinas | 10,9 | 13,0 | 14,5 | 17,9 | 17,4 | 18,9 | 19,6 | 20,4 | 23,5 |
| Santa Rita do Sapucaí | 4,2 | 8,4 | 10,6 | 15,7 | 14,9 | 17,5 | 18,4 | 19,1 | 20,2 |
| São João Del Rei | 5,8 | 8,9 | 10,8 | 15,5 | 14,9 | 17,4 | 18,2 | 18,9 | 20,0 |
| São Lourenço | 4,0 | 7,7 | 9,6 | 14,5 | 13,8 | 16,3 | 17,2 | 17,8 | 18,7 |
| São Sebastião do Paraíso | 4,5 | 10,1 | 12,4 | 17,1 | 16,3 | 18,6 | 19,3 | 19,9 | 22,2 |
| Sete Lagoas | 9,0 | 12,1 | 13,6 | 18,0 | 17,3 | 19,4 | 20,2 | 21,1 | 22,8 |
| Teófilo Otoni | 11,2 | 13,6 | 15,4 | 19,0 | 18,5 | 20,2 | 21,1 | 21,9 | 23,7 |
| Três Marias | 10,5 | 13,5 | 15,1 | 19,5 | 18,8 | 20,7 | 21,5 | 22,6 | 24,8 |
| Ubá | 8,4 | 11,5 | 13,4 | 17,9 | 17,4 | 19,9 | 20,8 | 21,5 | 22,7 |
| Uberaba | 6,9 | 12,8 | 15,2 | 19,4 | 18,7 | 20,5 | 21,1 | 22,1 | 25,0 |
| Uberlândia | 7,6 | 13,5 | 15,7 | 19,6 | 18,9 | 20,5 | 21,2 | 22,2 | 25,5 |
| Unaí | 12,5 | 14,9 | 16,3 | 19,7 | 19,2 | 20,6 | 21,2 | 22,1 | 24,9 |
| Varginha | 6,4 | 9,7 | 11,7 | 16,6 | 15,9 | 18,4 | 19,2 | 19,8 | 21,1 |
| Viçosa | 7,3 | 10,7 | 12,5 | 16,9 | 16,3 | 18,8 | 19,6 | 20,3 | 21,3 |

### Metarregressão

Tendo em vista a grande heterogeneidade entre as medidas de associação entre os modelos, foi realizada a introdução de variáveis socioeconômicas e geográficas no modelo para avaliação dessas medidas como explicativas da variação entre microrregiões (Tabela 4). O único teste de Wald com resultado significativo foi relacionado à altitude média da microrregião (p = 0,006), que revelou uma diminuição de 1,9% da medida de $I^2$ (58,1%), não a alterando de maneira importante.

Nesse sentido, ao reanalisar as medidas de associação em cada faixa de temperatura, considerando níveis de elevação nos percentis 10 e 90, pouca mudança pôde ser percebida, sendo todas as novas medidas pontuais contidas pelo IC95% das medidas do modelo base (Figuras 4b e 4c).

## Discussão

Os achados apresentados neste estudo demonstram uma relação não linear entre a temperatura mínima do ar e a incidência de dengue, sendo essa variável fator de risco e de proteção dependendo de sua intensidade: foi possível perceber que o frio moderado e extremo se comporta como fator protetivo, enquanto o calor moderado se associa como fator de risco. Esse tipo de relação é observada em experimentos laboratoriais que demonstram a temperatura como um dos principais fatores abióticos a influenciar a fisiologia e sobrevivência do mosquito *A. aegypti* e também do vírus da dengue [29,30,31].

Nesse sentido, estudos revelam que, em temperaturas do ar menores que 8ºC, as larvas do mosquito não se movem e morrem em poucos dias, enquanto o mosquito adulto se torna entorpecido e incapaz de se mover em temperaturas menores de 10ºC [30]. Por outro lado, o desenvolvimento, desde

**Figura 3**

Média de temperaturas mínimas das microrregiões de Minas Gerais, Brasil, 2010-2019.

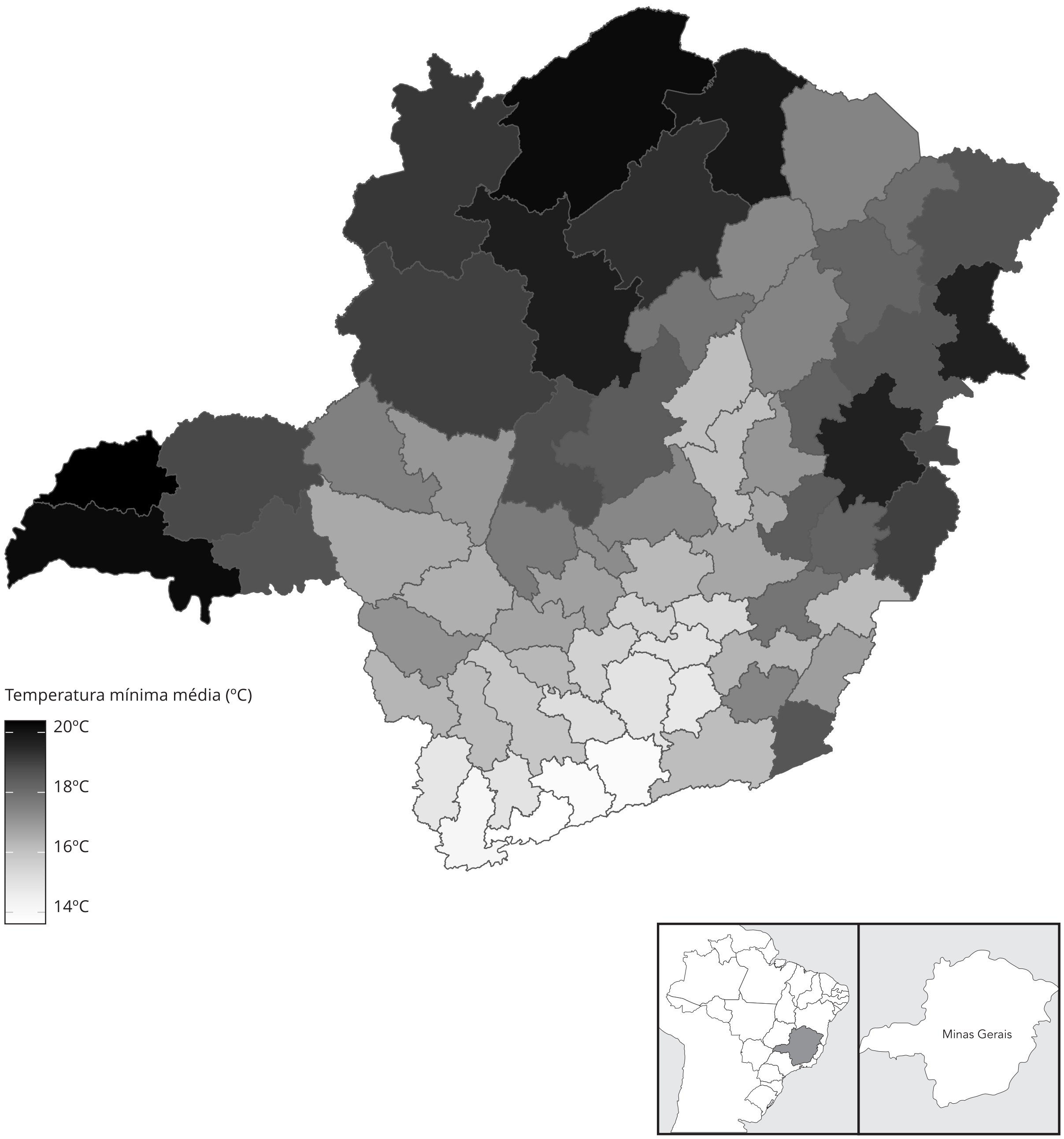

Fonte: Serviço de Mudança Climática da Copernicus [18].

**Tabela 3**

Riscos relativos (RR) por temperatura extremas e moderadas ajustados pelos percentis da altitude.

| Fonte | Classificação | Temperatura | RR | Limite inferior | Limite superior |
|---|---|---|---|---|---|
| Metanálise | Extremamente frias | 11,6 | 0,65 | 0,56 | 0,76 |
| | Moderadamente frias | 13,7 | 0,71 | 0,64 | 0,79 |
| | Moderadamente quentes | 21,2 | 1,15 | 1,07 | 1,24 |
| | Extremamente quentes | 22,4 | 1,10 | 0,99 | 1,22 |
| Altitude – percentil 10 | Extremamente frias | 11,6 | 0,62 | 0,54 | 0,72 |
| | Moderadamente frias | 13,7 | 0,69 | 0,63 | 0,77 |
| | Moderadamente quentes | 13,7 | 1,17 | 1,09 | 1,27 |
| | Extremamente quentes | 22,4 | 1,13 | 1,02 | 1,26 |
| Altitude – percentil 90 | Extremamente frias | 11,6 | 0,66 | 0,57 | 0,75 |
| | Moderadamente frias | 13,7 | 0,71 | 0,65 | 0,79 |
| | Moderadamente quentes | 13,7 | 1,15 | 1,07 | 1,24 |
| | Extremamente quentes | 22,4 | 1,10 | 0,99 | 1,22 |

**Figura 4**

Curva do risco relativo (RR) (25 dias de *lag*) de Minas Gerais, Brasil, e ajustada pelos percentis da altitude.

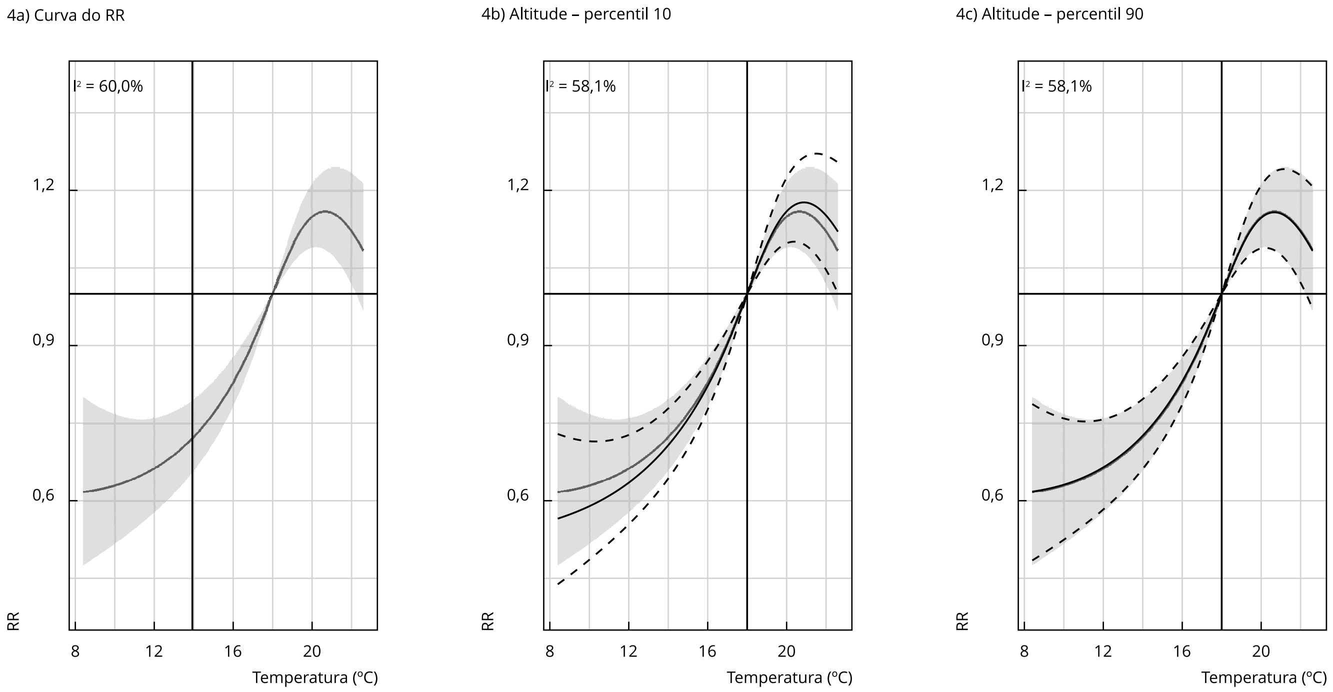

**Tabela 4**

Valor de p do teste de Wald a partir da adição de variáveis socioeconômicas e geográficas no modelo.

| Variáveis | Valor de p |
|---|---|
| População | 0,212 |
| PIB | 0,17 |
| PIB *per capita* | 0,157 |
| Altitude | 0,006 |
| Acesso à água tratada | 0,398 |
| Acesso à coleta de lixo | 0,347 |
| População urbana | 0,466 |
| Latitude | 0,684 |
| Longitude | 0,206 |

PIB: produto interno bruto.

a eclosão dos ovos até a fase adulta do mosquito, é mais curto em temperaturas do ar mais altas e em boas condições alimentares, mas seu desenvolvimento cessa acima de 36ºC. Assim, estima-se que a faixa de temperatura ideal para desenvolvimento do vetor seja de 16ºC a 34ºC [30].

Considerando a enorme influência do mosquito vetor na dinâmica da dengue, poderíamos supor que temperaturas mínimas extremamente frias levariam a um menor RR [31,32]. Por sua vez, esse risco aumentaria gradualmente com a elevação das temperaturas e retornaria a diminuir ao atingir temperaturas quentes extremas. No entanto, esse fenômeno foi apenas parcialmente verdadeiro em nosso estudo, uma vez que não foi possível observar um efeito significativo das temperaturas quentes extremas no RR do desfecho em nossa metanálise.

Outra variável de grande importância presente nos modelos apresentados e resumidos em nossa metanálise foi o tempo, mais especificamente o *lag*. A biologia do mosquito e a dinâmica epidemiológica da dengue demanda tempo: para que a doença se espalhe, é necessária a procriação, o desenvolvimento e o contato dos mosquitos com o vírus e, também, a incubação no vetor, a inoculação da dengue em um hospedeiro humano, a incubação nesse hospedeiro e a procura ao serviço de saúde pelo indivíduo [15,32]. Assim, o uso de modelos que analisam exposições e a influência delas em um acumulado de tempo permitem que efeitos de difícil interpretação sejam melhor detalhados e representados [15,33]. Dessa maneira, seguindo a linha de estudos que apontam a importância de variáveis como índices de seca e a própria temperatura mínima em escalas temporais maiores [10,13], nosso estudo demonstra como a temperatura mínima, uma variável facilmente observável, pode servir como um fator de alerta a profissionais de saúde em uma escala diária acumulada de tempo.

O tempo de desenvolvimento da larva do mosquito, sua sobrevivência e a reprodução do *A. aegypti* adulto são variáveis que dependem, de formas diferentes, da temperatura da água em que se encontram e da sua flutuação [29], sendo que a temperatura da água, intimamente relacionada à temperatura do ar [34], é inversamente proporcional ao tempo de desenvolvimento da eclosão do ovo do mosquito até a sua fase adulta [15]. Somado a isso, a temperatura do ar tem importância no que diz respeito ao tempo de incubação extrínseca da doença (no mosquito) e intrínseca (no ser humano) [32], influenciando esses períodos também de maneira não linear.

Até o momento, a maior parte dos estudos que analisam a relação entre a dengue e a temperatura do ar se utilizam de agregados de dados mensais ou semanais [10,13,35,36], que, apesar de mais facilmente encontrados, não produzem estimativas relacionadas a uma evolução de curto prazo da doença. Nosso estudo é um dos primeiros a utilizar variáveis diárias em sua análise.

No mais, outro importante resultado encontrado em nosso estudo foi o fato que, por mais que a temperatura mínima do ar tenha grande relação com a dengue, a forma que ela impacta a incidência é bastante diferente dependendo da microrregião de Minas Gerais. Foi constatado um alto grau de heterogeneidade das medidas de associação na metanálise, de modo que variáveis geográficas e socioeconômicas foram introduzidas no modelo definitivo para a avaliação, mas que, todavia, não causaram alterações relevantes no modelo. Isso leva à possibilidade de que variáveis explicativas de diferenças entre as microrregiões podem estar relacionadas a questões como efetividade de campanhas de educação e prevenção contra a dengue, tipo de urbanização, distribuição espacial de moradias, padrão de residência e mobilidade populacional, fenômenos já descritos na literatura com o potencial afetar a incidência da dengue [37,38]. Entretanto, essas variáveis são de difícil mensuração ou não estão disponíveis em todas as microrregiões analisadas.

O estudo de variáveis e métodos capazes de modelar e atualizar perspectivas relacionadas à dengue desempenha um papel crucial no entendimento da carga dessa doença a longo prazo e na preparação do sistema de saúde a curto e médio prazo. Especialmente quando se emprega uma variável climática como fator de predição, torna-se viável analisar as possíveis tendências da carga da doença nas predições atuais da emergência climática, o que pode fornecer perspectivas relacionadas ao seu impacto social e financeiro em diferentes cenários climáticos futuros [39,40]. No que diz respeito ao cenário de curto a médio prazo, a perspectiva de desenvolver um sistema de antecipação de surtos e epidemias tem despertado o interesse de epidemiologistas e pesquisadores [41,42,43]. A implementação de um sistema desse tipo possibilitaria a tomada de decisões relacionadas à prevenção e ao controle do vetor com base em dados, oferecendo maior previsibilidade e confiabilidade dos resultados. Para contextualizar essa necessidade, estima-se que de 40% a 72% da carga econômica da dengue esteja relacionada ao controle do vetor, mas não se sabe a real eficácia dessas ações, especialmente em locais onde os índices entomológicos não estão disponíveis [40,44,45]. Dessa forma, por não ter um sistema de apoio amplo e facilitado, essas ações podem ser menos eficazes e mais custosas.

A análise produzida neste estudo, com o uso de DLNM, adequados à dinâmica do vetor e da doença [15] e com amostra temporal de dez anos, mostra-se como um grande ponto forte. Esse tipo de modelo, usado em inúmeras pesquisas ao redor do globo para o estudo da dengue [10,13,35,36], e a subsequente metanálise produzem resultados com alto grau de confiança. O uso de dados diários também adiciona valor ao estudo, que além de demonstrar a importância da temperatura mínima como um fator explicativo para a incidência da dengue, ainda permite que modelos semelhantes possam gerar valores preditivos para gestores de saúde a níveis regionais, o que facilita a escolha de recursos de saúde coletiva e otimiza gastos.

É importante ressaltar que os dados utilizados neste estudo são provenientes de fichas de notificações individuais, tratadas pelo Ministério da Saúde brasileiro. Essa fonte é a mais atualizada e de melhor verossimilhança da realidade, com grande consistência relacionada aos campos utilizados em diversas regiões do país [46,47,48], por mais que ainda esteja sujeita a efeitos relacionados à subnotificação e má-classificação da doença. Além disso, em nosso estudo, optamos por utilizar a frequência das datas de notificação em vez de outra alternativa, como a data dos primeiros sintomas. Essa escolha foi baseada na grande consistência da variável [46] e comparabilidade com outros estudos, além da capacidade de controlar esse fator por meio da inclusão do dia da semana no processo de modelagem.

Por sua vez, a alta heterogeneidade apresenta a maior limitação do estudo, que foi a incapacidade de verificar variáveis explicativas para as diferentes medidas de associação entre as microrregiões. Esse fato leva à necessidade de novos estudos que procurem utilizar variáveis relacionadas à prevenção da dengue em análise. Ademais, o estudo também causou uma análise descritiva de todas as microrregiões, contudo não foi capaz de exibir medidas associativas da mesma forma, em virtude da pequena quantidade de casos em determinadas regiões. Mesmo assim, por mais que grande parte das microrregiões onde isso foi percebido sejam locais com pequenas populações, na região mais fria do estado, foram excluídas algumas cidades com número de habitantes semelhantes à de outras microrregiões incluídas. Esse fato levanta a hipótese de que um dos motivos para que essas regiões mais frias tenham tão poucos casos possa ser justamente o fato de observarem temperaturas não condizentes com a dinâmica do vírus e do vetor durante boa parte do ano. Esse fenômeno também pode ser observado em regiões do Sul do Brasil, em que a incidência da dengue é muito menos intensa [49].

## Conclusão

Os resultados apresentados neste estudo demonstram que, nas microrregiões de Minas Gerais, a temperatura mínima do ar é um fator protetivo em temperaturas moderadas e extremamente frias, enquanto, em temperaturas quentes moderadas, ela passa a ser um fator de risco. Contudo, essa variável não é suficiente para explicar toda a variabilidade encontrada entre as microrregiões, sugerindo a presença de outros fatores não mensurados que afetam a dinâmica da doença. Portanto, estudos que incluam variáveis relacionadas a campanhas de saúde, tipo de urbanização e densidade habitacional – ausentes neste estudo – podem ser úteis para compreender melhor os fatores que influenciam a incidência da dengue em Minas Gerais.

## Colaboradores

J. P. M. Gomes contribuiu com a concepção do estudo, análise e interpretação dos dados, redação e revisão crítica; e aprovou a versão final. I. M. Ribas contribuiu com a concepção do estudo, análise e interpretação dos dados, redação e revisão crítica; e aprovou a versão final. P. A. R. Valadares contribuiu com a concepção do estudo, análise e interpretação dos dados, redação e revisão crítica; e aprovou a versão final. L. S. Jardim contribuiu com a concepção do estudo, análise e interpretação dos dados, redação e revisão crítica; e aprovou a versão final. M. C. Nogueira contribuiu com a concepção do estudo, análise e interpretação dos dados, redação e revisão crítica; e aprovou a versão final. C. C. M. Ferreira contribuiu com a concepção do estudo, análise e interpretação dos dados, redação e revisão crítica; e aprovou a versão final. A. S. A. Watanabe contribuiu com a concepção do estudo, análise e interpretação dos dados, redação e revisão crítica; e aprovou a versão final. L. C. M. Ferreira contribuiu com a concepção do estudo, análise e interpretação dos dados, redação e revisão crítica; e aprovou a versão final.

## Informações adicionais

ORCID: João Pedro Medeiros Gomes (0000-0001-5576-2481); Igor Magaton Ribas (0000-0002-0029-4462); Pedro Augusto Rosa Valadares (0000-0001-7019-4056); Lucas Santos Jardim (0000-0001-6132-455X); Mário Círio Nogueira (0000-0001-9688-4557); Cássia de Castro Martins Ferreira (0000-0002-6070-7257); Aripuanã Sakurada Aranha Watanabe (0000-0003-0875-2269); Letícia de Castro Martins Ferreira (0000-0002-2916-4477).

## Agradecimentos

Ao apoio da Universidade Federal de Juiz de Fora com uma bolsa de iniciação científica.

## Abstract

*Air temperature is a climatic factor that affects the incidence of dengue, with effects varying according to time and space. We investigated the relationship between minimum air temperature and dengue incidence in Minas Gerais, Brazil, and evaluated the influence of socioeconomic and geographic variables on this relationship. This is a time series study with analysis conducted in three distinct stages: modeling using a distributed lag non-linear model, meta-analysis of models obtained, and meta-regression with geographic and socioeconomic data. Minimum temperature was a protective factor at extreme cold temperatures (RR = 0.65; 95%CI: 0.56-0.76) and moderate cold temperatures (RR = 0.71; 95%CI: 0.64-0.79), and a risk factor at moderate hot temperatures (RR = 1.15; 95%CI: 1.07-1.24), but not at extreme hot temperatures (RR = 1.1; 95%CI: 0.99-1.22). Heterogeneity of the models was high ($I^2$ = 60%), which was also observed in meta-regression. Moderate and extreme cold temperatures have a protective effect, while moderate hot temperatures increase the risk. However, minimum air temperature does not explain the variability in the region, not even with the other variables in meta-regression.*

*Dengue; Climate; Time Factors; Temperature*

## Resumen

*La temperatura del aire es un factor climático que afecta la incidencia del dengue, con efectos que varían según el tiempo y el territorio. Investigamos la relación entre la temperatura mínima del aire y la incidencia de la enfermedad en Minas Gerais, Brasil, y evaluamos la influencia de variables socioeconómicas y geográficas en esta relación. Se trata de un estudio de serie temporal cuyo análisis se realiza en tres etapas distintas: modelación mediante el uso de* distributed lag non-linear model *(modelos distributivos no lineales con retraso), metaanálisis de los modelos obtenidos y metarregresión con datos geográficos y socioeconómicos. La temperatura mínima fue un factor de protección ante temperaturas extremadamente frías (RR = 0,65; IC95%: 0,56-0,76) y moderadas (RR = 0,71; IC95%: 0,64-0,79) y factor de riesgo en temperaturas de calor moderado (RR = 1,15; IC95%: 1,07-1,24), pero no en extremo (RR = 1,1; IC95%: 0,99-1,22). La heterogeneidad de los modelos fue alta ($I^2$ = 60%), y esta medida no se modificó en la metarregresión. Las temperaturas frías moderadas y extremas tienen un efecto protector, mientras que las temperaturas moderadamente altas aumentan el riesgo. Sin embargo, la temperatura mínima del aire no explica la variabilidad de la región, ni siquiera con las demás variables en metarregresión.*

*Dengue; Clima; Factores de Tiempo; Temperatura*

Recebido em 12/Mai/2023
Versão final reapresentada em 09/Nov/2023
Aprovado em 24/Nov/2023
